# Recombinantly Expressed Chimeric Fibers Demonstrate Discrete Type-Specific Neutralizing Epitopes in the *Fowl Aviadenovirus E* (FAdV-E) Fiber, Promoting the Optimization of FAdV Fiber Subunit Vaccines towards Cross-Protection *in vivo*

**DOI:** 10.1128/spectrum.02123-21

**Published:** 2022-01-19

**Authors:** Anna Schachner, Carlotta De Luca, Sarah Heidl, Michael Hess

**Affiliations:** a Christian Doppler Laboratory for Innovative Poultry Vaccines, University of Veterinary Medicine, Vienna, Austria; b Clinic for Poultry and Fish Medicine, Department for Farm Animals and Veterinary Public Health, University of Veterinary Medicine, Vienna, Austria; University of Sussex

**Keywords:** fowl adenovirus, fiber, cross-protection, epitopes, neutralization, inclusion body hepatitis

## Abstract

Vaccines against inclusion body hepatitis in chickens are complicated by the involvement of antigenically diverse fowl adenovirus types. Though immunization with fiber protein confers robust protection, type specificity of fiber antibodies is an obstacle for the desired broad coverage. In this study, we utilized information on multiple linear epitopes predicted in the *Fowl Aviadenovirus E* (FAdV-E) fiber head (knob) to develop chimeric fibers with an exchange between two serotypes’ sequences, each containing proposed epitopes. Two consecutive segments pertaining to amino acid positions 1 to 441 and 442 to 525/523 in the fibers of FAdV-8a and -8b, types of *Fowl Aviadenovirus E* that cause inclusion body hepatitis, were swapped reciprocally to result in novel chimeras, crecFib-8a/8b and crecFib-8b/8a. crecFib was indistinguishable from monospecific recombinant fibers in its eactivity with different FAdV antisera in Western blotting. However, contrary to the results for monospecific fibers, crecFib induced cross-neutralizing antibodies against both serotypes in chickens. This demonstrates three nonidentical epitopes in the FAdV-E fiber, the conserved epitope detected in Western blotting and at least two epitopes participating in neutralization, being type specific and located opposite residue position 441-442. Furthermore, we supply conformational evidence for a site in the fiber knob with accessibility critical for neutralization. With such an extended neutralization spectrum compared to those of individual fibers, crecFib was anticipated to fulfill and even extend the mechanistic basis of fiber-mediated protection toward bivalent coverage. Accordingly, crecFib, administered as a single-antigen component, protected chickens simultaneously against challenge with FAdV-8a or -8b, demonstrated by up-to-complete resistance to clinical disease, prevention of target organ-related changes, and significant reduction of viral load.

**IMPORTANCE** The control of inclusion body hepatitis, a disease of economic importance for chicken production worldwide, is complicated by an etiology involving multiple divergent fowl adenovirus types. The fiber protein is principally efficacious in inducing neutralizing and protective antibodies in vaccinated chickens; however, it faces limitations due to its intrinsic type specificity for neutralization. In this study, based on an *in silico*-guided prediction of multiple epitopes in the fowl adenovirus fiber head’s loops, we designed chimeric proteins, swapping N- and C-distal fiber portions, each containing putative epitopes, between divergent types FAdV-8a and -8b. In *in vitro* and *in vivo* studies, the chimeric fiber displayed extended properties compared to those of individual monotype-specific fibers, allowing the number, distribution, functionality, and conformational bearings of epitopes of the fowl adenovirus fiber to be characterized in more detail. Importantly, the chimeric fiber induced cross-neutralizing antibodies and protective responses in chickens against infections by both serotypes, promoting the advancement of broadly protective subunit vaccination strategies against FAdV.

## INTRODUCTION

Knowledge about adenoviral antigenicity is predominantly derived from human adenoviruses, from the pioneer work on the major determinants by Norrby (1) to a range of studies on epitope identification. More recently, such studies have also advanced beyond the immunodominant antigen, hexon, and started to involve novel *in silico* approaches (2–4). Such information is widely unavailable for adenoviruses outside the genus *Mastadenovirus*, despite holding clinical applications, not only for certain veterinary areas but also for potential development of new vectorization platforms. In the field of avian adenoviruses, several early works on typing by investigation of antigenic relationships contributed to a principal understanding of the relevant domains (5). However, studies to identify and characterize individual epitopes of bird adenoviruses are still exceptionally rare.

The group-reactive antigen (designated α, residing in the hexon), noted shortly after its discovery as principally discriminatory between mammalian and avian adenoviruses, is reflected in the historical taxonomic division of avian adenoviruses into groups I, II, and III, subsequently recognized as members of different genera (6, 7). Especially because of the diversity of *Fowl Aviadenovirus* (FAdVs), the classical adenoviruses of chickens belonging to the genus *Aviadenovirus*, a need for further differentiation shifted focus to the main type-specific (ε) determinant harbored by the hexon. This antigen with its neutralizing properties serves for serological distinction of 12 types (FAdV-1 to -8a and -8b to -11) within the recognized species *Fowl Aviadenovirus A* to *Fowl Aviadenovirus E* (FAdV-A to FAdV-E).

However, with the fiber as an additional carrier of different reactivities (subgroup and type specific, referred to as δ and λ determinants), this has recently exposed a possible pitfall of stand-alone hexon typing. Based on the discovery of natural FAdV recombinants with exchanges between hexon and fiber of different types, fiber was shown as probably the second-most-potent contributor to type-specific neutralization besides hexon (8).

The type-specific component of fiber has already proven amenable to use in refining serological detection of FAdVs, advancing from whole-virus-based enzyme-linked immunosorbent assays (ELISAs) with broad-spectrum detection to type differentiation with isolated fiber proteins as coating antigens (9–11). Furthermore, fiber has been repeatedly confirmed as a subunit vaccination antigen with high protective efficacy against different FAdV-induced pathologies in chickens. However, a dilemma arises for certain fiber subunit vaccines, due to the fact that neutralization is the mechanistic basis of fiber-induced protection, while fiber antibodies are intrinsically type specific (12). This severely limits fiber as an antigen for tackling multitype disease complexes, importantly in the case of inclusion body hepatitis (IBH), caused by three major antigenically discriminate clusters (FAdV-2/11 of species FAdV-D and FAdV-8a and FAdV-8b of species FAdV-E) (13). Moreover, neutralizing antibodies (NAbs) to fiber were absent in some studies and in others supported as a correlate of protection (12, 14–16), but there are indications for a more important role in FAdV species with a singular fiber (rather than those with dual fibers), underlining the relevance of neutralization for IBH. And yet, the only *in vitro* functional data on fiber epitopes are based on an exceptional model with two fiber genes, FAdV-4 (of species FAdV-C) (17), and contrary to findings *in vivo*, those data support that all serotypes’ fibers possess a neutralizing epitope.

In this study, we used *in silico*-generated information on multiple epitopic sites in the fiber head domain for engineering a sequence exchange between the two discrete serotypes FAdV-8a and -8b, anticipated to result in new full-length fiber proteins with mixed-epitope compositions. The antibody subsets induced by such chimeric fibers *in vivo* resolved the coexistence of multiple epitopes, along with their discriminate specificities and functionality. In accordance with this, a challenge experiment proved the newly developed chimeric fiber efficacious for simultaneous control of the major serotypes causing IBH, which was previously unattainable with native fibers.

## RESULTS

### *In silico* design and recombinant expression of crecFib constructs.

**FIG 1:**
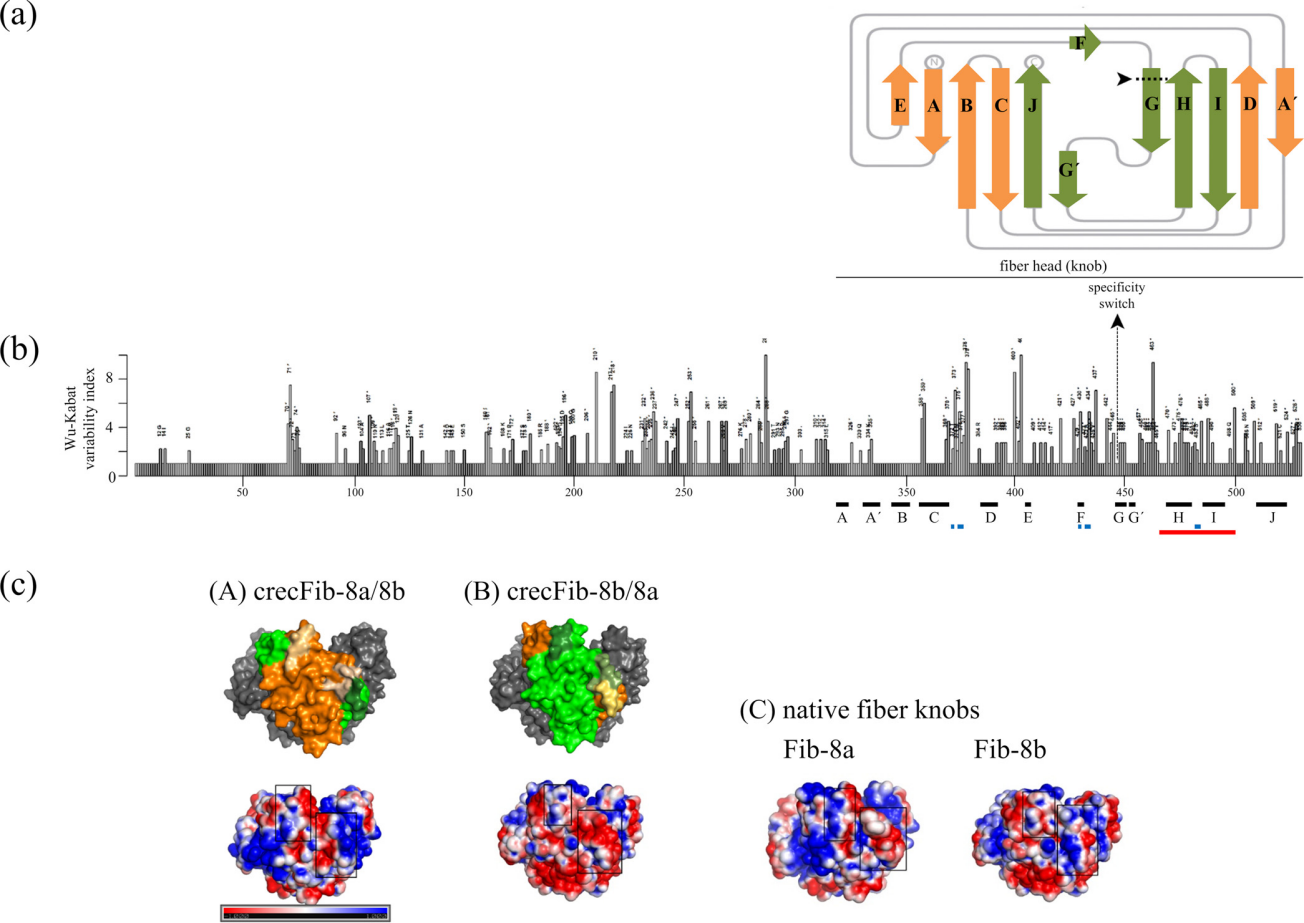
(a) The crecFib knob domain, represented schematically as a 2-dimensional ribbon model (adapted from reference 32). Arrows represent β-strands (designated A to J, in linear order from the N to the C terminus of the knob sequence) connected by lines representing the intervening loops. Structures encoded by sequences of discriminate serotypes are shown in different colors opposite the chimeric exchange site (the “specificity switch,” indicated by the dashed line at the left end of the G strand). (b) Coordinates of β-strands of the fiber knob (black bars), candidate epitopes (blue bars, epitopes predicted by DiscoTope 2.0; red bar, a previously reported epitope [17]), and the specificity switch, annotated in a Wu-Kabat plot of the FAdV-E fiber sequence. The degree of variability, indicated by the bar size for each residue position, has been calculated as the Wu-Kabat index from available fibers representing all types of the species (*n* = 29). Amino acid positions of the multiple sequence alignment are annotated along the horizontal axis. Candidate epitopes conform well with less tightly conserved residues, while the specificity switch is located at an intraspecies consensus site. (c) Comparisons of the two crecFib knobs (8a/8b and 8b/8a) by similarity modeling of the amino acid sequences (A and B) with their predicted surface electrostatics in the panels underneath, with the molecule shown in side view. In the top panels, only one chain of the fiber trimer is colored, with orange tones for the sequence portion derived from FAdV-8a and green tones for the sequence portion from FAdV-8b; muted tones indicate the three epitopes predicted in this study. The same epitopes are highlighted with black squares in the electrostatic surface charge images with color transitions from red (negative charge) to blue (positive charge) and are compared to the corresponding sites in the cognate native knobs of FAdV-8a and -8b (C).

*In silico* epitope analysis of the strain TR59 and strain 746 fiber knobs suggested sites that, based on homology modeling, were assigned to the CD loop (amino acids G.SSD and N.PTG), the β-strand F/FG loop (amino acids V.DANP and I.DASS), and the HI loop of the knob (amino acids QSQ and RSQ) (Fig. 1a). According to 3-dimensional models created on the basis of the chimeric knobs’ sequences, all predicted epitopes were localized externally, fulfilling the criterion of surface accessibility, as well as apically on the molecule (Fig. 1c), corresponding to the side in HAdVs that participates in virus-host interaction (18). Furthermore, the two sites in the β-strand F/FG loop and HI loop, though noncontiguous in the primary sequence, were conformationally in contact, revealing a coherent surface patch formed by residues encoded N and C distally from the chimeric junction.

Both chimeric proteins, designated crecFib-8a/8b and crecFib-8b/8a, were successfully recovered from the soluble fraction of infected Sf9 cells, as confirmed by bands with the appropriate monomer size in Western blots (identical to the control proteins used for the Western blots shown in Fig. 3). The yields of purified chimeric fibers were approximately 13.4 mg (crecFib-8a/8b) and 14.8 mg (crecFib-8b/8a) per liter of Sf9 cell culture.

### Immunogenicity and *in vitro* reactivity spectrum of crecFib. (i) crecFib antibody induction detected by ELISA.

**FIG 2:**
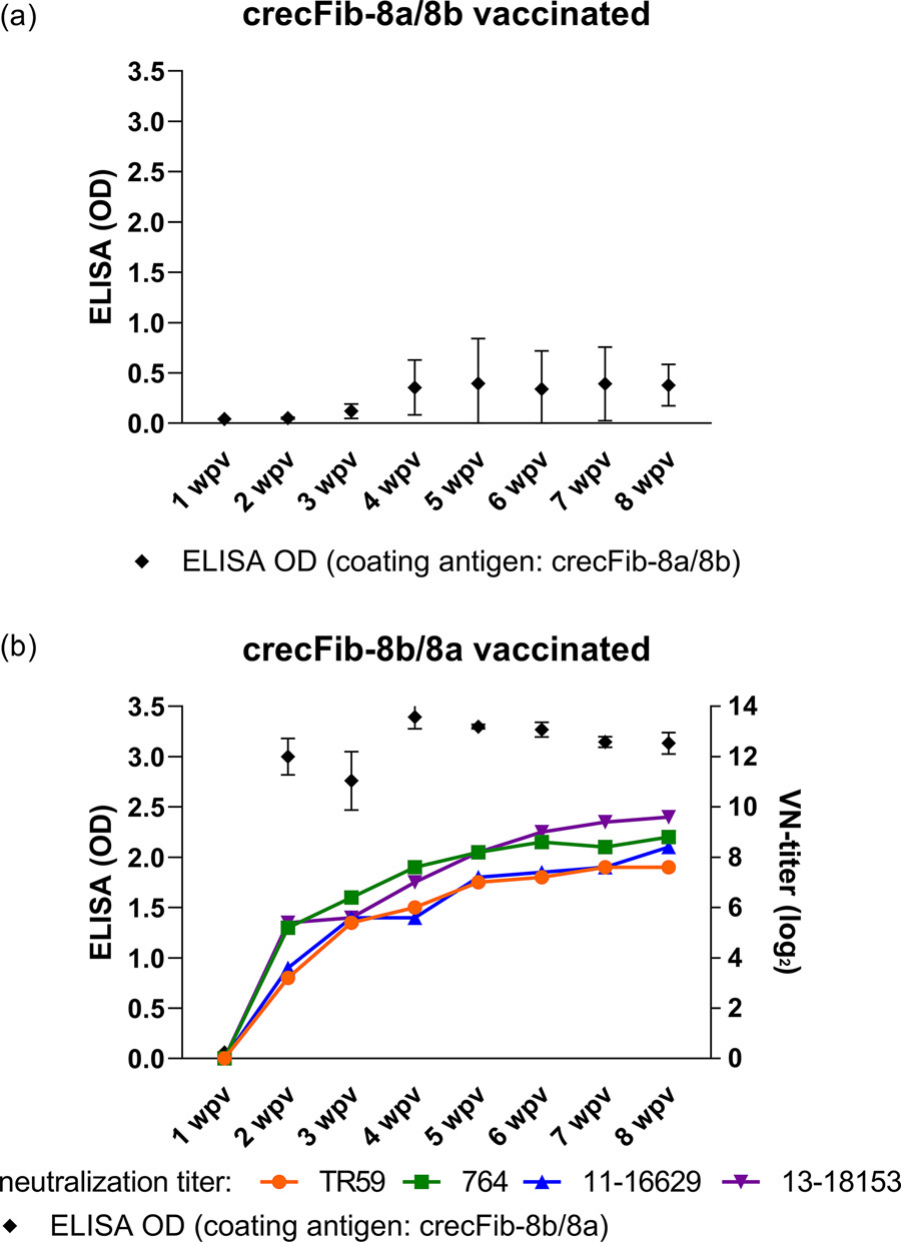
Antibody development following immunization with crecFib constructs (*n* = 5 birds for each construct). The header of each panel indicates the construct used for immunization, which also corresponds to the coating antigen of the ELISA for determination of weekly ODs from 1 to 8 weeks post vaccination (wpv). Mean neutralization titers, if detectable, are plotted with the respective colors for the viruses against which the sera were tested (including the chimeric template and the challenge strains of this study from FAdV-8a and -8b). Error bars show standard deviations.

Based on the homologous-antigen ELISA, sera from birds immunized with crecFib-8a/8b showed a flat increase in optical density (OD) magnitude, with only sporadic occurrence of ODs of >1 in a single individual at three successive time points from 5 to 7 weeks post vaccination (wpv). Sera from all other birds did not exceed an OD of 0.5 at any time point during an 8-week monitoring period (Fig. 2a). In contrast, sera from birds immunized with the reverse-order crecFib-8b/8a developed sharp rises in ODs in the homologous-antigen ELISA as early as 2 wpv, when sera from 5/5 birds already presented with ODs close to 3 (mean OD ± standard deviation, 3.00 ± 0.18), independent of the antigen dose (50 versus 100 μg). The crecFib-8b/8a ODs remained at a constant level in all 5 individuals until the end of the monitoring period at 8 wpv, with mean weekly ODs in the range of 2.76 ± 0.29 to 3.39 ± 0.12 (Fig. 2b).

### (ii) Neutralizing activity of crecFib antisera.

No neutralizing activity was detected in any of the birds immunized with crecFib-8a/8b, including the individual with an indicative ELISA OD (Fig. 2a).

Among crecFib-8b/8a antisera, neutralization was first present at 2 wpv, with all birds showing low to moderate titers (4 to 7 log_2_) against at least one of the constitutive types (Fig. 2b). In fact, 4/5 individuals had already developed bilateral NAbs against both types at this time point. The neutralizing titers of the crecFib-8b/8a antisera continuously increased during the monitoring period; from 5 wpv onwards, all birds had titers of ≥6 log_2_ against the complete set of tested strains, with one bird reaching the maximum of the measured range (14 log_2_) against FAdV-8b. On an individual-bird level, the neutralizing responses showed an overall balanced distribution, with similar titers against both types, regardless of whether the corresponding virus was a template (reference) or field strain.

Immunofluorescent staining of a virus neutralization (VN) setting, which directly compared crecFib-8b/8a antiserum side by side with antifiber antisera against Fib-8a or Fib-8b on the same microtiter plate, demonstrated that only the chimeric serum could efficiently inhibit the infection of both FAdV-8a (strain TR59) and FAdV-8b (strain 764) *in vitro* (Fig. S1 in the supplemental material). While the monospecific Fib-8a and Fib-8b antisera exerted neutralizing activity against their cognate serotypes at titers similar to those of the crecFib antiserum, neither of them could inhibit the opposite serotype.

### (iii) Recognition of fibers in Western blotting.

**FIG 3:**
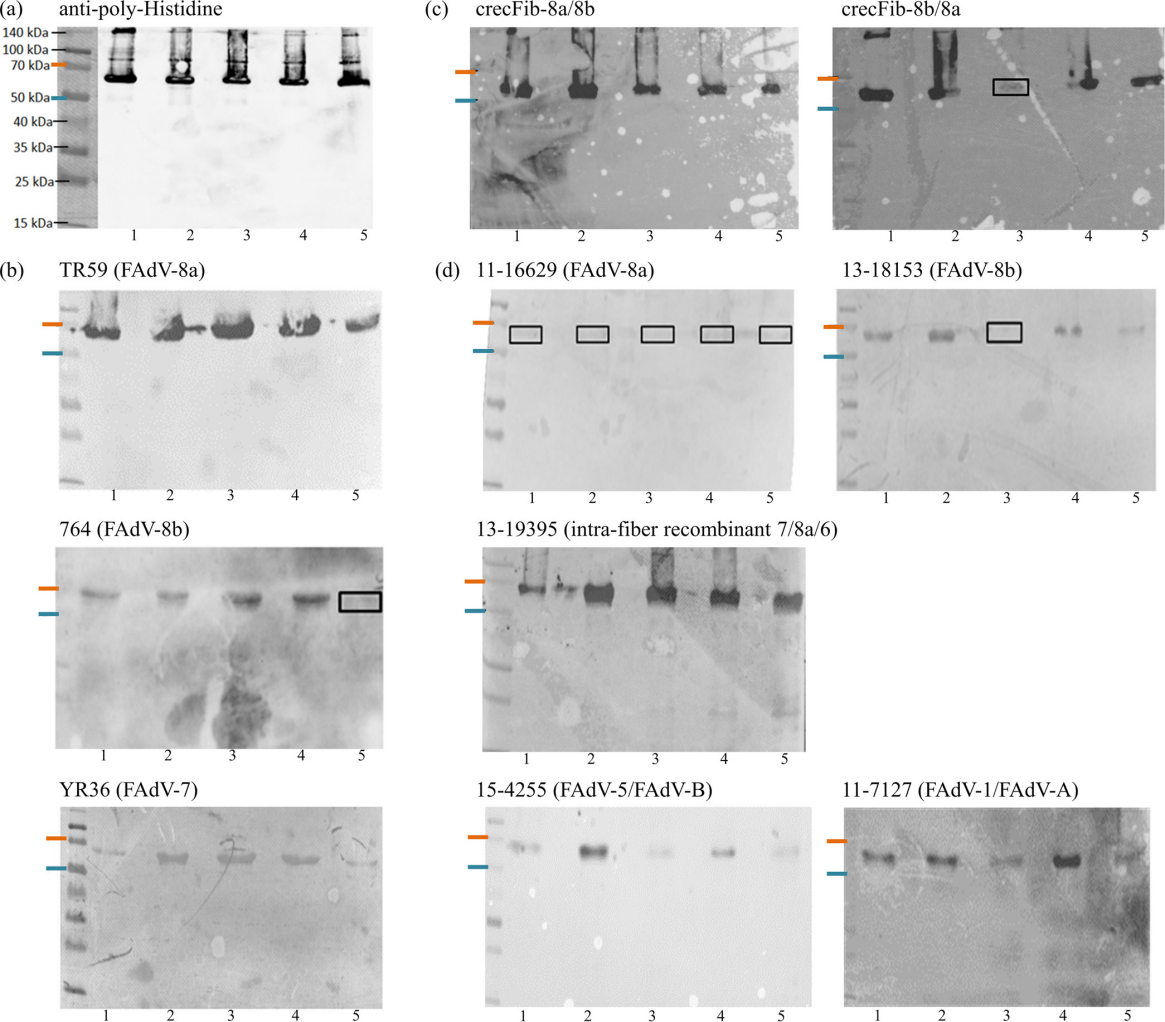
Western blot analysis to assess the reactivities of crecFib constructs side by side with monospecific control fibers from types FAdV-8a, FAdV-8b, and FAdV-7 (all from species FAdV-E). The order of antigens is the same on all membranes as labeled for the first membrane: 1, Fib-8a; 2, Fib-8b; 3, crecFib-8a/8b; 4, crecFib-8b/8a; 5, Fib-7. However, each membrane was incubated with a different antiserum, specified in the header of each membrane, with the corresponding serotype in parentheses. Molecular weight sizes are indicated by the standard in the first lane in panel a; in all subsequent blots, only the relevant 50 kDa-to-70 kDa range is marked. Signals that are present but difficult to distinguish from the background are boxed. (a) Confirmation of monomeric fibers via the polyhistidine tag. (b) Detection of crecFib by antisera raised against the constitutive strains from FAdV-8a and -8b and FAdV-7 as the outlier type. (c) Detection of crecFib by antisera from birds vaccinated with the same or the reverse-order construct. CrecFib antisera also recognized all monospecific control fibers. (d) Detection of crecFib and control fibers by antisera against field isolates, including the two challenge strains of this study, a naturally recombinant strain, and exemplary strains from species other than FAdV-E with either one (FAdV-B) or two (FAdV-A) fiber genes.

All recombinant fibers investigated, independent of their genetic background and their monospecific or chimeric composition, were detected by immune sera representing the complete spectrum of FAdV types, defined by fiber specificity (Fig. 3).

Mutual recognition was even possible between reaction partners with differential fiber expression (fiber-1 and fiber-2 of FAdV-A and FAdV-C versus the singular fiber of the remaining FAdV species) and between chimeric counterparts with the reverse template order (e.g., crecFib-8b/8a antiserum was able to recognize crecFib-8a/8b) (Fig. 3b to d).

Of note, no differences were noted between antisera raised against live (native) FAdV and inactivated, adjuvant-formulated virus preparations, as well as subunit-directed antisera, in their ability to recognize the fiber monomer in immunoblots (data not shown as identical signals were obtained for the same protein when incubated with any type of antiserum).

### Protection study 1: protective efficacy of crecFib-8a/8b and crecFib-8b/8a.

**FIG 4:**
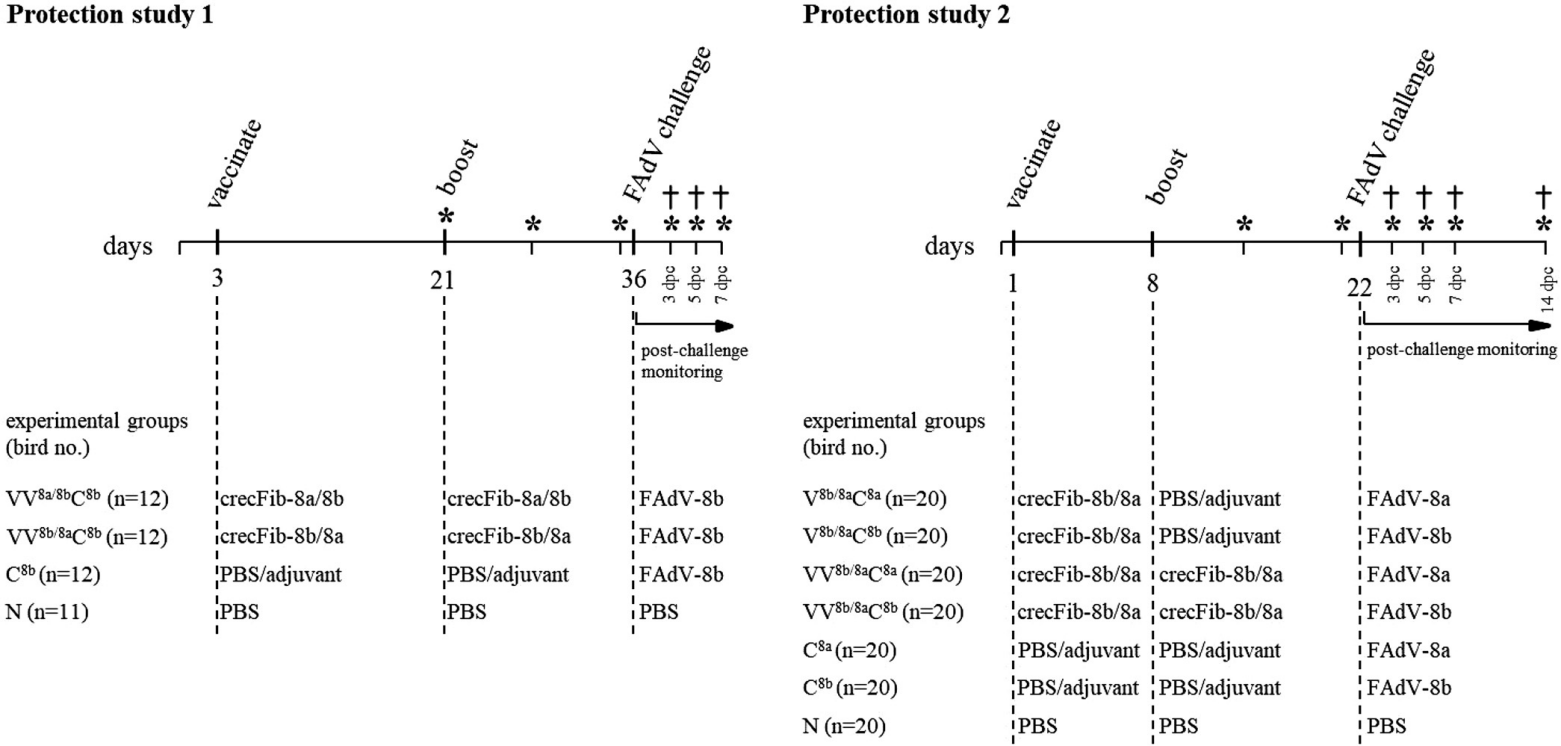
Experimental design of protection studies 1 and 2. The procedures (vaccination, booster, and challenge) carried out on birds are indicated on the timelines (as days of life and days post challenge [dpc]). Individual treatment schemes of each group are specified below. Asterisks indicate blood sampling, and cross symbols indicate sequential killings, with necropsy and organ sampling.

An overview of the *in vivo* experimental designs is provided in Fig. 4. A group designated VV^8a/8b^C^8b^, according to the treatments carried out, was vaccinated twice with crecFib-8a/8b at 3 and 21 days of life, followed by FAdV-8b challenge at 36 days of life (15 days post booster [dpb]). A second group, designated VV^8b/8a^C^8b^, was vaccinated at the same time points with the reverse-order crecFib-8b/8a and then also challenged with FAdV-8b. The challenge control, mock vaccinated with a phosphate-buffered saline (PBS)/adjuvant mixture prior to FAdV-8b challenge, was designated C^8b^, and the negative control, administered only sterile PBS at each occasion, was designated N.

**FIG 5:**
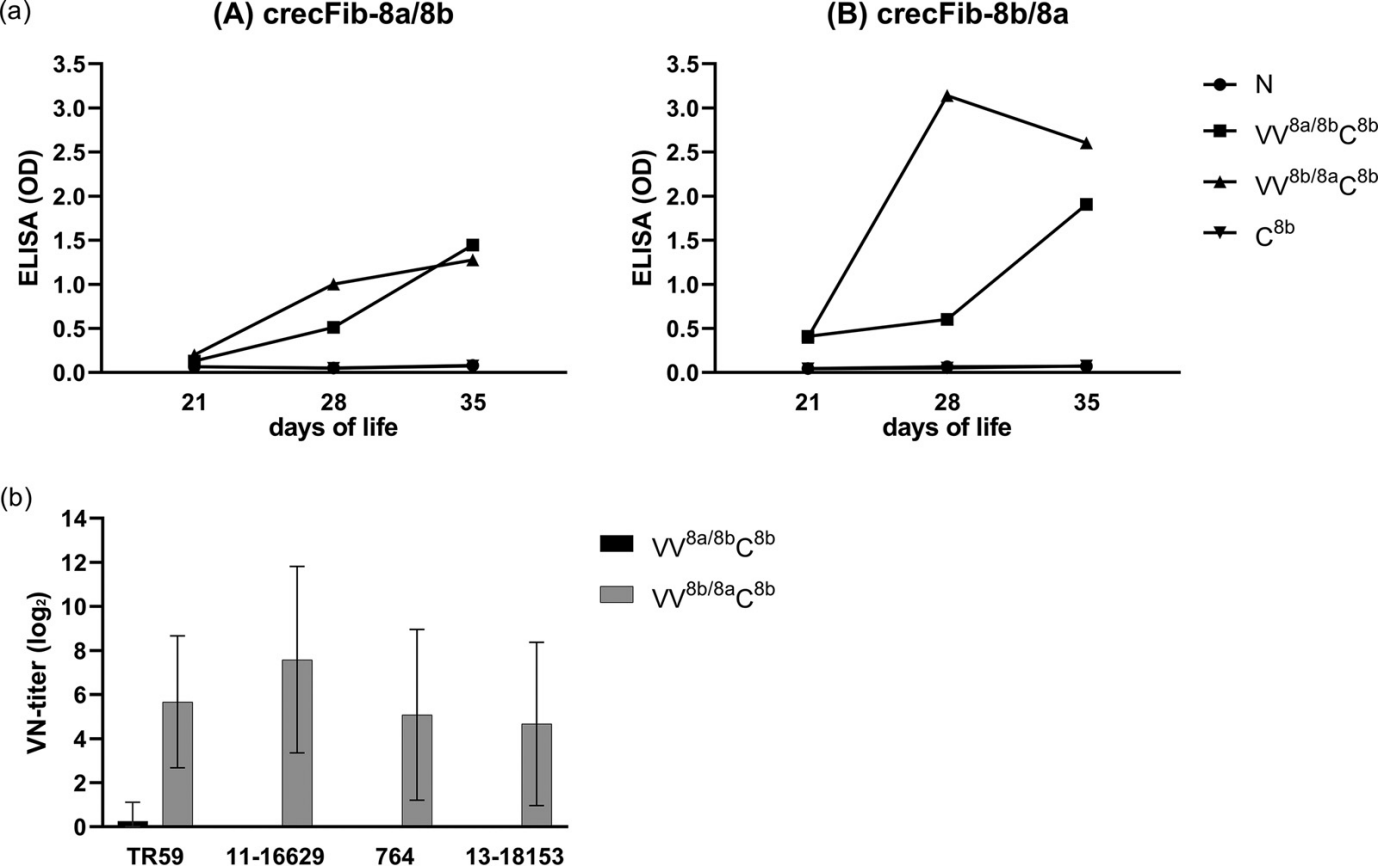
Prior challenge antibody development in protection study 1 (prime-boost vaccination with crecFib-8a/8b or crecFib-8b/8a). (a) Mean OD values for each treatment (groups receiving the same treatment were pooled), measured by crecFib-8a/8b ELISA (A) and crecFib-8b/8a ELISA (B) at 21 (18 dpv), 28 (7 dpb), and 35 (14 dpb) days of life. (b) Neutralizing antibody titers at 35 days of life (14 dpb, immediately prior to challenge) against FAdV-8a (strains TR59 and 11-16629) and FAdV-8b (strains 764 and 13-18153). Error bars show standard deviations.

The onset of vaccine-induced antibody development was not detected by ELISA until after the booster (7 dpb, 28 days of life), and only at a low level, indicating the beginning of a rise, in birds of the VV^8a/8b^ group (mean OD, 0.51 ± 0.65), while peak detectable levels were already reached in the VV^8b/8a^ group (3.14 ± 0.73) (Fig. 5a). In comparison, sera from birds of the N group and the C^8b^ group (prior to challenge) never exceeded an OD of 0.07 ± 0.01. Immediately before challenge (14 dpb), sera from birds of the VV^8a/8b^ group reached moderate ODs (1.45 ± 0.95), but none of the sera exhibited neutralizing activity (VN titers of ≤8 log_2_). In sera from the VV^8b/8a^ group, the OD levels remained rather constant (2.60 ± 0.85), and with the exception of a single bird, all sera exhibited neutralizing activity, ranging from low titers against at least one of the tested serotypes (in 3 birds) to titers at the highest end of the measured range (14 log_2_) against both serotypes (FAdV-8a and -8b reference and challenge viruses) in the remaining birds. As a general trend, higher titers (and earlier onset, based on those sera with only unilateral neutralization) were noted against FAdV-8a; in addition, NAbs against FAdV-8b never diverged by more than 3 titer levels between reference and challenge strains, while NAbs against FAdV-8a diverged by up to 5 titer levels, with more pronounced reactivity against the challenge than the reference strain (Fig. 5b).

**FIG 6:**
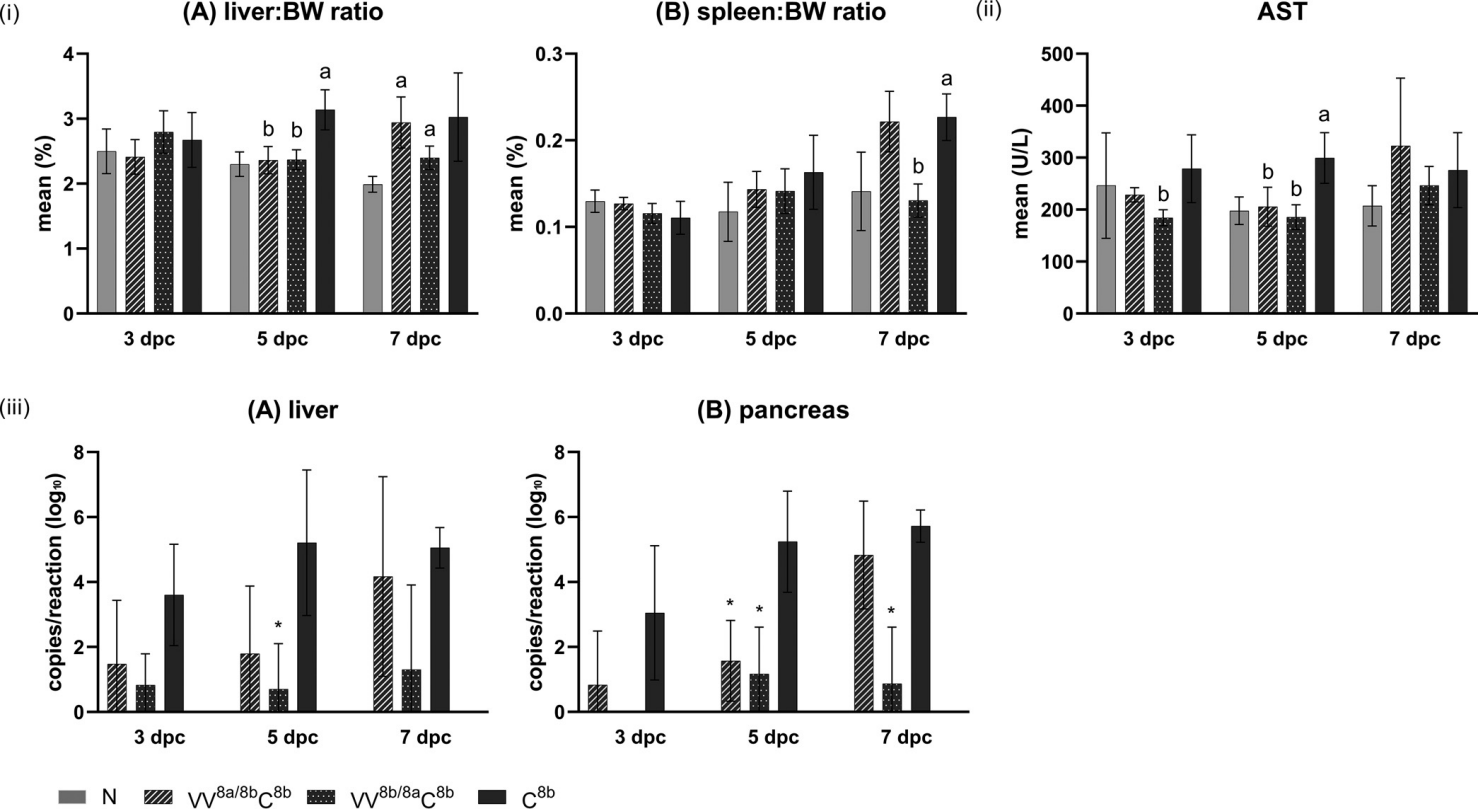
Endpoints of protection, evaluated after FAdV-8b challenge in protection study 1 (at 3, 5, and 7 dpc). (i) Organ-body weight (BW) ratios for liver (A) and spleen (B). Lowercase letters above bars indicate significant differences (a, versus negative control; b, versus challenge control). (ii) Plasma AST (a, significantly different versus negative control; b, significantly different versus challenge control). (iii) Viral loads in liver (A) and pancreas (B). Significant differences versus challenge control are indicated with asterisks (*P ≤ *0.05). Error bars show standard deviations.

Following challenge, mild depression (inappetence, huddling, and ruffled plumage) was recorded in two birds of the C^8b^ group at 4 to 5 dpc. During necropsy at this time point, the same individuals had significantly increased liver/body weight (BW) ratios. An increase of liver/BW ratios was also recorded in birds of the the VV^8a/8b^C^8b^ and VV^8b/8a^C^8b^ groups, but the occurrence was delayed, at 7 dpc (Fig. 6i). The C^8b^ group additionally experienced a significant increase of the spleen/BW ratio (7 dpc), which was absent in the VV^8b/8a^C^8b^ group, whereas it occurred at a moderate but not significant level in the VV^8a/8b^C^8b^ group.

The highest plasma aspartate transaminase (AST) values were recorded in birds in the C^8b^ group at 3 and 5 dpc, being significant at 5 dpc, whereas the plasma AST values in birds in the VV^8a/8b^C^8b^ and VV^8b/8a^C^8b^ groups remained comparable to the values in birds in the N group (Fig. 6ii). At 7 dpc, plasma AST values were not distinguishable between the groups anymore.

A reduction of hepatic viral loads versus the loads in birds in the C^8b^ group was noted in birds in both the VV^8a/8b^C^8b^ and VV^8b/8a^C^8b^ groups at all time points investigated, with the lowest values throughout and significance at 5 dpc in the VV^8b/8a^C^8b^ group (Fig. 6iii). In the pancreas, viral DNA was completely absent in birds in the VV^8b/8a^C^8b^ group at 3 dpc and significantly reduced at 5 and 7 dpc. In birds in the VV^8a/8b^C^8b^ group, the pancreatic viral load was significantly reduced only at 5 dpc.

### Protection study 2: broad protective efficacy of crecFib-8b/8a applying different vaccination regimens.

A prime-boost vaccination regimen at 1 day old and 7 days postvaccination (dpv) was administered to two groups, one of which was challenged at 22 days of age with FAdV-8a (designated VV^8b/8a^C^8a^) and the other with FAdV-8b (VV^8b/8a^C^8b^). Additionally, the results of the experiment were compared to those of a single-vaccination regimen administered at 1 day old (groups V^8b/8a^C^8a^ and V^8b/8a^C^8b^). Mock-vaccinated challenge control groups were included for each challenge type (C^8a^ and C^8b^), as well as a negative-control (N) group receiving PBS at time points analogous to the prime-boost regimen.

**FIG 7:**
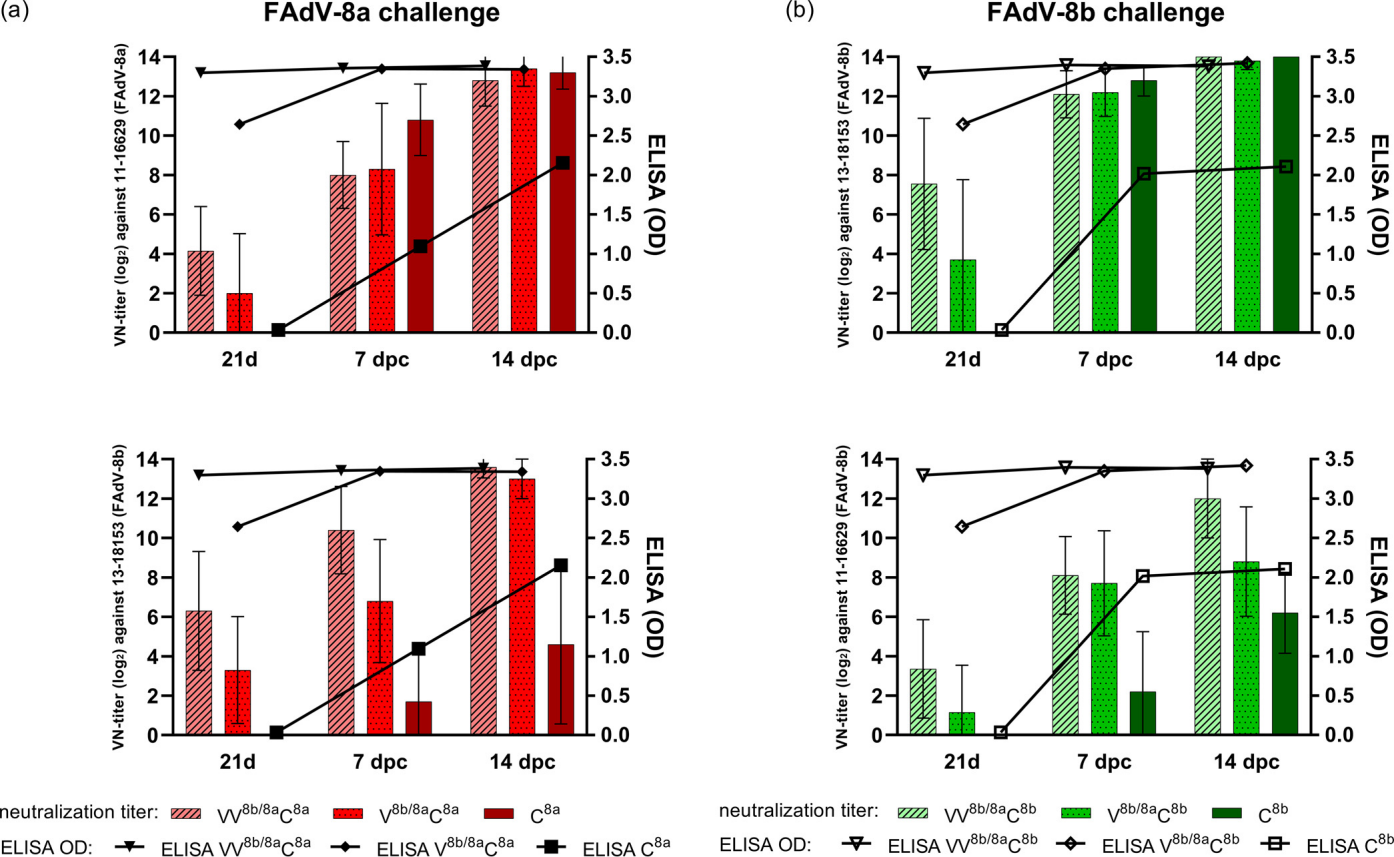
Antibody development (ELISA and VN test) in protection study 2 (comparing the V^8b/8a^ and VV^8b/8a^ regimens against challenge with either FAdV-8a or -8b) immediately prior to (21 days), and after (7 and 14 dpc) challenge. Only groups that received the same challenge were compared among each other, with a summary of data for FAdV-8a-challenged birds (strain 11-16629) in panel a and of FAdV-8b challenged birds (strain 13-18153) in panel b. Top, neutralization against the virus type used for challenge; bottom, neutralization against the respective heterologous virus type. Error bars show standard deviations.

At 21 days of life, immediately prior to challenge, birds from groups receiving each vaccination regimen had developed antibody levels detectable by ELISA. However, sera from birds in the prime-boost group (VV^8b/8a^) had higher ODs, with only 1/40 individuals having an OD of <3 (mean OD, 3.3 ± 0.1), while sera from birds receiving the single-vaccine regimen (V^8b/8a^) had overall lower, more unevenly distributed titers (2.65 ± 0.79) (Fig. 7). Post challenge and irrespective of the challenge virus type, the ODs between sera from the V^8b/8a^ and VV^8b/8a^ groups were not distinguishable anymore, as they reached (and then remained at) the highest measurable endpoint. In contrast, sera from nonvaccinated challenge control birds showed a steady incline of titers to 2.15 ± 1.02 until 14 dpc (C^8a^) or an abrupt rise to 2.02 ± 0.95 at 7 dpc, thereupon remaining stable (C^8b^).

In 36/40 VV^8b/8a^ birds, prechallenge Abs had neutralizing activity against both FAdV-8a and -8b, while the remaining four birds showed only unilateral neutralizing activity against one of the serotypes. In comparison, single-vaccinated V^8b/8a^ birds showed a more infrequent presence of prechallenge NAbs, with 11/40 birds having unilateral NAbs and 7 birds having a complete absence of NAbs. Furthermore, the titer levels were lower in V^8b/8a^ birds than in VV^8b/8a^ birds, although the regimens were similar in eliciting stronger neutralization against FAdV-8a (3.28 ± 3.22 log_2_ in V^8b/8a^ birds versus 5.58 ± 2.00 log_2_ in VV^8b/8a^ birds against FAdV-8a and 2.88 ± 2.44 log_2_ in V^8b/8a^ birds versus 4.30 ± 3.13 log_2_ in VV^8b/8a^ birds against FAdV-8b).

The FAdV-8a challenge resulted in a relatively abrupt increase of NAbs in naive birds (C^8a^), reaching higher levels than in any of the vaccinated groups at 7 dpi (Fig. 7a). In VV^8b/8a^C^8a^ birds, overall higher titers against FAdV-8b than FAdV-8a were found, while the reverse trend was seen in V^8b/8a^C^8a^ birds; by 14 dpc, birds of all three groups reached comparable mean titers against FAdV-8a. FAdV-8b challenge induced mean titers in naive birds (C^8b^) that were similar to those induced by FAdV-8a challenge at 7 dpc (10.70 ± 1.34 log_2_) but did not significantly exceed the mean titers of vaccinated birds (V^8b/8a^C^8b^ and VV^8b/8a^C^8b^) (Fig. 7b). Although V^8b/8a^C^8b^ and VV^8b/8a^C^8b^ birds continued to develop NAbs against FAdV-8a, the mean titers against FAdV-8b were still always higher.

Of note, individual challenged birds of the C^8a^ and C^8b^ groups also showed a certain cross-neutralization of the heterologous viral type, though those were exclusively birds with peak homologous titers (12 to >14 log_2_).

As a benchmark for clinical effects following challenge, mild depression was recorded in one (C^8a^) and four (C^8b^) birds between 2 and 5 dpc, and an additional dead bird at 4 dpc in the C^8b^ group. Among all birds of the vaccinated groups, only one case of transient depression occurred at 2 to 3 dpc, less surprisingly in an individual of the V^8b/8a^C^8b^ groups that lacked prechallenge NAbs.

**FIG 8:**
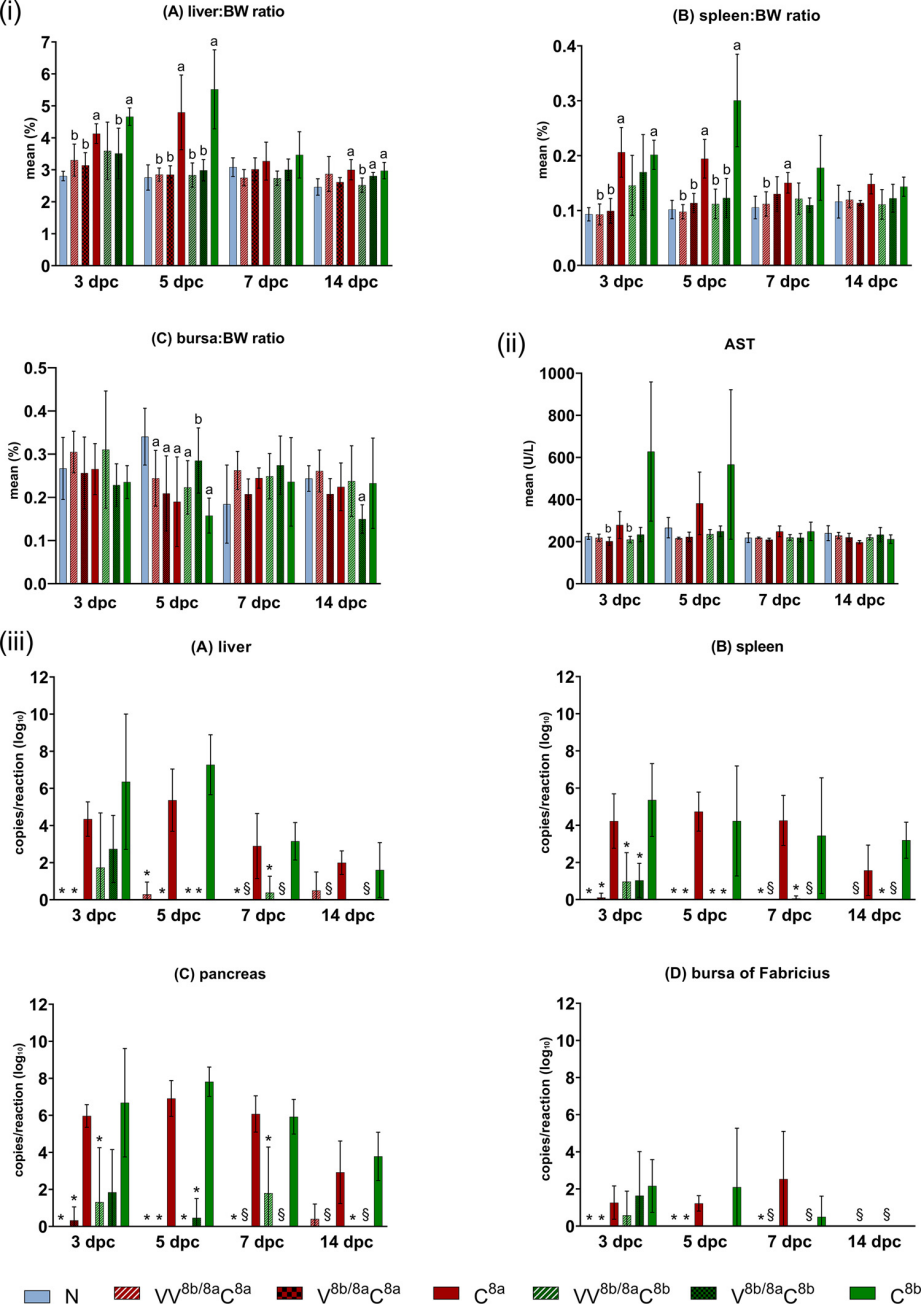
Endpoints of protection in protection study 2 (evaluated at 3, 5, 7, and 14 dpc). The groups are designated with V or VV according to single or prime-boost regimen with crecFib-8b/8a, followed by C with the respective challenge virus indicated (either FAdV-8a or FAdV-8b); N designates the negative control. (i) Organ-body weight (BW) ratios for liver (A), spleen (B), and bursa of Fabricius (C). Lowercase letters above bars indicate significant differences (a, versus negative control; b, versus the corresponding challenge control). (ii) Plasma AST levels (a, significantly different versus negative control; b, significantly different versus challenge control). (iii) Viral loads in liver (A), pancreas (B), spleen (C), and bursa of Fabricius (D). Significant differences versus the corresponding challenge control are indicated with asterisks (*P ≤ *0.05). Data exclude the single-vaccination regimen at 7 and 14 dpc due to low sample size (marked with §). Error bars show standard deviations.

The mean liver/BW and spleen/BW ratios were most affected at 3 and 5 dpc, with significant increases in birds in both challenge control groups (Fig. 8i). Despite levelling off, this effect still persisted for the spleen at 7 dpc in the C^8a^ group and for the liver at 14 dpc in both the C^8a^ and C^8b^ groups. In contrast, the liver/BW and spleen/BW ratios in the vaccinated/challenged groups remained comparable to those in the negative control (N) group throughout the whole experiment, except for the liver/BW ratio in the V^8b/8a^C^8b^ group at one time point (14 dpc). Bursa/BW ratios were most affected at 5 dpc, with a significant reduction in all groups versus the ratio in the N group, except for the V^8b/8a^C^8b^ group, in which the same trend was shifted to 14 dpc.

In addition, birds in the C^8a^ and C^8b^ groups had the highest plasma AST values of all groups from 3 to 7 dpc; significant differences were found compered to the plasma AST values in two of the vaccinated groups (VV^8b/8a^C^8b^ and V^8b/8a^C^8a^) (Fig. 8ii). Furthermore, vaccinated groups exhibited consistently lower viral loads in target organs compared to their challenge controls, while vaccination even prevented detectable infection at several time points (Fig. 8iii). With few exceptions, liver, pancreas, and spleen samples of birds in the C^8a^ and C^8b^ groups were positive at all time points (only one liver at 7 dpc and the pancreas and spleen from another bird at 14 dpc in the C^8a^ group were negative, as well as two livers at 14 dpc from birds in the C^8b^ group); in contrast, viral DNA was detected only in one bird’s liver at 5 dpc and another bird’s liver and pancreas at 14 dpc in the VV^8b/8a^C^8a^ group, with viral loads significantly reduced from 3 to 7 dpc. Similar results were achieved in the V^8b/8a^C^8a^ group between 3 and 5 dpc, with only one bird’s pancreas and spleen testing positive at the earliest time point (3 dpc). In the VV^8b/8a^C^8b^ group, the mean viral loads were significantly reduced at 5 to 7 dpc in the liver and at all time points in the pancreas and spleen; in fact, all of the target organs remained even completely negative at 5 and 14 dpc. In the V^8b/8a^C^8b^ group, significant reductions of viral loads occurred only in the spleen at 3 to 5 dpc and in the liver and pancreas at 5 dpc. The bursa of Fabricius was the organ with the lowest mean viral load, although positive results were still found in all but 2 samples in the C^8a^ group and in 4, 3, and 1 sample(s) in the C^8b^ group from 3 to 7 dpc. Of all vaccinees, only three individuals, having no prechallenge NAbs and challenged with FAdV-8b, were positive at 3 dpc. At 14 dpc, viral DNA was generally not detectable in the bursa of Fabricius anymore.

## DISCUSSION

Most of the knowledge about epitopes in *Fowl Aviadenovirus* (FAdV) capsid proteins is still deduced from the heavily investigated human adenoviruses (HAdVs), lacking, however, a directly comparable antigenic constellation. Particularly the fiber, despite its increasing popularity as a candidate subunit vaccine against FAdVs, is less resolved in regard to its antigenic properties. Complicating the situation, adenoviral fibers show individual variations within their common morphological framework. This includes the number and type of fibers present on the capsid, but also variations in the surface accessibility of residue sites, with consequences for receptor tropism and immunity (3, 19).

In the FAdV-8b fiber (species FAdV-E), two B cell epitopes were previously identified (20). Located in the fiber shaft, those epitopes were, however, nonneutralizing, which conforms with similar reports from HAdVs (21). In contrast, we recently found evidence for at least 2 discrete neutralizing epitopes in the FAdV-E fiber, mapping inside or near the knob, based on hybrid neutralization of a wild-type strain with a fiber gene recombined between two serotypes (8). As the only further information about neutralizing fiber epitopes derives from FAdV-4 (17), a member of the species FAdV-C, which peculiarly encodes two fiber proteins in two separate genes, differences between the studied models were expected. Notably, however, the FAdV-E fiber sequences analyzed in the present study had a predicted epitope in the same location as that of the FAdV-4 fiber-2 reported for type-specific *in vitro* neutralization. This, alongside two other *in silico*-identified candidate epitopes in the FAdV-E knob, prompted us to design a crosswise sequence exchange between fibers of FAdV-8a and -8b, the two FAdV-E affiliate serotypes causing inclusion body hepatitis (IBH) (22).

The resulting protein, termed crecFib, was amenable to confer simultaneous protection against each viral type included in the chimeric construct, as demonstrated by up to complete prevention of clinical disease and significant limitation (or, in some instances, delay) of effects of virus in target organs, as well as organic and metabolic damage.

Sera from chimera-vaccinated birds neutralized both virus types, in contrast to sera against monospecific control fibers, which strictly neutralized virus of the same serotype. This reinforces the results of our previous study in which the FAdV-8a fiber failed to protect chickens against FAdV-8b challenge, showing that fiber is a monovalent vaccine antigen due to the requirement of functionally neutralizing Abs for protection (12). Though the reliance on NAbs seems to pertain more to those types causing IBH (i.e., types with one fiber gene), considering controversial findings with fiber vaccines against hepatitis-hydropericardium syndrome (HHS) (FAdV-4, the type with two fiber genes), a greater practical interest arises for IBH due to (i) its multitype etiology and (ii) the resistance of the involved types to cross-neutralization by fiber Abs.

Of the two recombinant chimeras tested in this study, only one combination, crecFib-8b/8a, was adequately immunogenic, as shown by robust ELISA antibody titers, the presence of neutralizing Abs, and up to complete protection following challenge.

With three-dimensional models of the crecFib knobs made available, we speculate on a possible role of structural differences between the two constructs, specifically in a region that evoked our interest because it merges residues from opposite sides of the chimeric switch, each accounting for separate epitopes in the linear sequences. Besides discriminate topologies, the corresponding region showed overall inverted surface electrostatics with positively charged residues in the weakly immunogenic crecFib-8a/8b, as opposed to a predominantly negative potential in the superior crecFib-8b/8a. Charge conversion can substantially influence the immunogenicity of antigens, sometimes resulting from only a single mutation (23, 24). It is therefore possible that the changed residue constellations at the point of contact between sequences from different template strains in chimeras altered their surface charge, with consequences for presentation to the immune system.

The conformational nature of this region alongside local changes of structure and electrostatics in crecFib proteins with different immunogenicities provides intriguing hints toward a major neutralizing epitope. The importance of conformational epitope(s) for functional neutralization by the FAdV fiber is also supported by an earlier work, in which non-hexon-specific MAbs against FAdV-1 (species FAdV-A) full virus with neutralizing activity (identifying them with some certainty as fiber Abs) could not detect denatured viral proteins (25). This also resembles the situation in HAdVs, where fiber-directed NAbs preferentially recognize conformational epitopes and trimeric fiber (26, 27).

Aside from the structural effects discussed, our data on the immunogenicities of chimeras with reciprocally oriented sequences are similar to those of another study, conducted with HAdV fibers, where the outcome of *in vitro* reactivities of chimeric knobs varied due to different locations of the dominant neutralization epitope depending on the serotype (3). Although this hypothesis remains to be investigated for FAdVs, our results would indicate the location of the neutralizing epitope to be anywhere to the right in the FAdV-8a fiber, as opposed to being to the left in the FAdV-8b fiber, relative to the exchange site G441-R442, rendering crecFib-8b/8a the only, but very powerful, antigen attainable by such means.

Despite its inability to elicit a humoral response, the reverse-order crecFib-8a/8b still showed good reactivity with diverse antifiber antisera in Western blotting. As Western blotting, unlike fiber ELISA and VN test, was also the only method that allowed cross-detection of fibers, this pinpoints an epitope that is broadly shared but accessible only in the denatured protein. We propose that this epitope, which is distinct from the epitope(s) inducing NAbs and does not contribute to protection, resides N distally in the tail, the most strongly conserved of all fiber domains, similarly to the broadly reactive epitope identified in HAdV fiber monomers (28, 29).

In conclusion, we resolved distinct antigenic interfaces, together with their putative locations in the FAdV fiber, having different structural and functional properties that can be exploited for the design of chimeric proteins. *In vivo*, chimeric fibers were confirmed to extend the protective spectrum of conventional fiber subunits. Allowing diverse neutralizing epitopes to be condensed into a single-antigen component for broad coverage, chimeric fibers are a suitable vaccination strategy to address the advancing emergence of FAdV types worldwide.

## MATERIALS AND METHODS

### *In silico* design for recombinantly expressed chimeric fibers.

The FAdV fiber open reading frame was divided into an amino (N)- and a carboxy (C)-distal segment, amplified separately from different template strains and fused seamlessly via Gibson Assembly cloning to reconstitute a novel, full-length fiber, herein referred to as “crecFib” (Fig. 1a and b). As a result, crecFib constructs contain a crossover between heterologous sequences of serotypes FAdV-8a and -8b at the junction of N- and C-distal segments. The junction (termed a “specificity switch”) was engineered at consensus residue positions of amino acids 441-442 in the pairwise sequence alignment of the FAdV-8a and FAdV-8b fibers, mapping inside the proposed fiber head (knob) domain. Accommodating *in silico*-predicted epitopes to both sides, this strategy was anticipated to combine the antigenic specificity of both constitutive serotypes into a single antigen. By engineering the specificity switch inside the knob, we also sought to maintain sequence integrity at the presumed shaft-knob boundary, which represents an important element for trimerization of the fiber (30).

Candidate epitopes were (i) inferred from a previously reported epitope in fiber-2 of FAdV-4 (species FAdV-C) (17) and (ii) *in silico* prediction with DiscoTope 2.0 software (31), whereby the closest related fiber knob for which a molecular model is currently available, fiber-2 of FAdV-1 (species FAdV-A) reference strain CELO (32), served for homology modeling. Positional homologies between members of different types were determined by multiple sequence alignments created with MegAlign software (DNAStar, Madison, WI, USA). Homology modeling was also used for assigning structural domains (tail, shaft, and knob) of the FAdV fiber based on existing information (33).

### Cloning and expression of crecFib constructs.

Based on the FAdV reference strains selected as expression templates, the above-defined N- and C-distal fiber segments were amplified using primer pairs featuring overhangs with the flanking sequence of the EheI-/StuI-digested pFastBac expression vector (Invitrogen, Vienna, Austria) and between the two segments themselves. Detailed information on the cloning strategy is provided in Table S1. Ligation into the linearized pFastBac vector was performed with the Gibson Assembly master mix (NEB, Ipswich, MA) according to the manufacturer’s instructions. Two constructs were generated for each chimeric combination, with reciprocal specificity order, designated crecFib-8a/8b and crecFib-8b/8a accordingly.

Correct insertion of the segments into the vector was confirmed by Sanger sequencing across the multiple-cloning site (LGC Genomics, Berlin, Germany).

Recombinant proteins were expressed in Spodoptera frugiperda Sf9 cells and purified via polyhistidine tags on affinity chromatography columns as described previously (14), and their concentrations determined by Bradford assay (Thermo Fisher Scientific, Vienna, Austria).

### Assessment of the immunogenicity and *in vitro* reactivity spectrum of crecFib.

Specific-pathogen-free (SPF) chickens (Valo BioMedia GmbH, Osterholz-Scharmbeck, Germany), hatched and housed at our facilities, were immunized intramuscularly (i.m.) with crecFib-8a/8b (*n* = 5, 3-day-old) or crecFib-8b/8a (*n* = 5, 14-day-old). Three birds of each group received 50 μg and two birds 100 μg of recombinant protein mixed 1:1 with GERBU adjuvant P (GERBU Biotechnik GmbH, Heidelberg, Germany). Postimmunization sera were collected at weekly intervals for parallel monitoring by enzyme-linked immunosorbent assay (ELISA) and virus neutralization test.

The procedures on experimental birds were discussed and approved by the institutional ethics committee and licensed by the Austrian government according to the approval requirements of the Animal Experiments Act 2012 (34) (license number GZ 68.205/0006-V/3b/2019).

Additional immune sera for comparative purposes were sourced from sample collections of previously published studies or recruited under animal trial license numbers GZ 68.205/0217-WF/V/3b/2016 and GZ 68.205/0006-V/3b/2019 (summarized in Table S2).

Briefly, these sera were derived from SPF chickens injected with whole virus or immunized i.m. with monospecific FAdV fibers (i.e., recombinantly expressed fibers based on the sequence of a singular serotype). Whole virus for antiserum production was 3-fold plaque purified and characterized at least by partial analysis of the hexon and fiber genes but in most cases by full-genome sequencing and cross-neutralization test, as described previously (35). Whenever available, our test setting included sera against inactivated, adjuvanted FAdV (prepared with 1% formaldehyde, administered in a 1:1 mixture with GERBU adjuvant) and live virus, in order to assess a possibly differential recognition of denatured antigen in the immunoblot.

### crecFib-based ELISA.

Sera from birds immunized with each type of crecFib were tested on ELISA plates coated with the corresponding crecFib, following the protocol described by Feichtner et al. (10).

### Virus (cross-)neutralization (VN) test.

Neutralizing antibodies (NAbs) in sera were determined in a microtiter assay on primary chicken embryo liver (CEL) cells. Serial 2-fold serum dilutions (1:8 to 1:16,384) were incubated with 100 50% tissue culture infective doses (TCID_50_) of virus. The crecFib antisera were tested against the FAdV-8a and -8b reference (expression template) strains and two field isolates from each serotype which served as challenge strains in the protection study. For comparative purposes, antifiber antisera with monotype specificity (Fib-8a and Fib-8b serum) were included in certain settings. Additionally, each microtiter plate included positive (cells plus virus) and negative (cells only) control wells. After 5 days at 37°C in 5% CO_2_, the wells were investigated for cytopathic effect (CPE), with the titer defined by the highest serum dilution inhibiting CPE.

### Immunofluorescence staining.

Following a virus cross-neutralization test against FAdV-8a and -8b reference strains, as described above, the cells were fixed by adding ice-cold methanol for 5 min. Following serial washing steps and blocking with 3% bovine serum albumin (BSA) for 1 h, in-house-generated polyclonal rabbit anti-FAdV antiserum (diluted 1:500 in PBS) was added to each well overnight. After removal of the rabbit antiserum and washing, the plates were incubated with 1:200-diluted Alexa Fluor 488-conjugated donkey anti-rabbit IgG (Invitrogen, Life Technologies, Carlsbad, CA, USA) in the dark for 1 h. After another wash, cells were stained with 1:1,000 DAPI (4′6-diamidino-2-phenylindole solution; Roche Diagnostics GmbH, Vienna, Austria) for 5 min, subjected to a final wash, and covered with PBS.

Internalization of viral particles was examined and documented with a Zeiss Axiovert 200 M fluorescence microscope (Zeiss, Jena, Germany) coupled to a Flexacam C1 camera and Leica Application Suite X (LAS X) (Leica Microsystems GmbH, Wetzlar, Germany).

### Western blotting.

The crecFib constructs were screened side by side with the most closely related, in-house-expressed monospecific fibers (Fib-8a of strain TR59, Fib-8b of strain 764, and Fib-7 of strain YR36, the reference type representatives of FAdV-E) using polyclonal immune sera. In order to minimize variations in the ratios of reactants, the concentration of recombinant protein loaded per lane was adjusted to 7.5 μg, and detection sera with similar ELISA titers (2.5 ≤ OD ≤ 3.0) against recombinant fiber of the homologous type and, if applicable, neutralization titers in the range of 11 to 12 log_2_ were used. Briefly, recombinant purified proteins were separated by 12% SDS–PAGE and transferred onto BioTrace polyvinylidene difluoride (PVDF) transfer membrane (Pall, Vienna, Austria) with the Trans-Blot Turbo transfer system (Bio-Rad, Vienna, Austria). After blocking with 3% (wt/vol) skim milk, membranes were incubated separately with polyclonal sera, preabsorbed with 1% Sf9 cell powder, and diluted 1:2,000. As a control for the presence and size of monomeric fibers, one membrane was incubated with antipolyhistidine antibody (Sigma-Aldrich, Vienna, Austria). Following incubation with secondary rabbit anti-chicken IgG-horseradish peroxidase (HRP) (Sigma-Aldrich, Vienna, Austria), or goat anti-mouse IgG(H+L)-HRP (Bio-Rad, Vienna, Austria) for controls, and intermediate washes, blots were developed with Clarity Western ECL substrate (Bio-Rad, Vienna, Austria). Visualization was performed with the ChemiDoc Imager (Bio-Rad, Vienna, Austria).

### Protection studies with crecFib constructs.

Two vaccination-challenge trials were performed to sequentially address whether (i) crecFib constructs confer *in vivo* protection and (ii) crecFib-induced protection is amenable for broad coverage of the inclusion body hepatitis complex. An overview of both experimental designs is summarized in Fig. 4. The corresponding procedures on birds were discussed and approved by the institutional ethics committee and licensed by the Austrian government (license numbers GZ 68.205/0156-V/3b/2019 and GZ 68.205/0215-V/3b/2019).

### (i) Protection study 1: protective efficacy of crecFib-8a/8b and crecFib-8b/8a.

In the first study, two groups of SPF broiler chickens (*n* = 12) were prime-boost vaccinated with either crecFib-8a/8b or the reverse-order crecFib-8b/8a, followed by challenge with FAdV-8b in each case. SPF broilers were obtained from Animal Health Service (Deventer, The Netherlands) and housed in separate isolator units (HM2500; Montair, The Netherlands). Vaccination consisted of 50 μg of the respective crecFib formulated in a 40% (wt/vol) antigen-oil-based adjuvant phase, administered i.m., while challenge was carried out i.m. with 106.2 TCID_50_ FAdV-8b (strain 13-18153). Further groups served as a challenge control group, injected with a PBS/adjuvant mixture instead of vaccination, and a negative-control group, administered only sterile PBS according to the same scheme. Blood was collected weekly from booster until challenge and at 3, 5, and 7 days post challenge (dpc). Four birds per group were killed and submitted to necropsy at 3 and 5 dpc, analogous to the remaining birds at 7 dpc. Endpoints of protection included clinical signs, assessed daily in the time period post challenge, organ-body weight (BW) ratios for liver and spleen, the aspartate transaminase (AST) content in plasma as previously described (36), and viral load quantification in liver and pancreas by a quantitative PCR (qPCR) protocol adapted from Günes et al. (37).

### (ii) Protection study 2: broad protective efficacy of crecFib-8b/8a applying different vaccination regimens.

In this setting, we proceeded with only one of the chimeras, crecFib-8b/8a, this time testing its protective efficacy against challenge with both viral types of interest (FAdV-8a or FAdV-8b). Additionally, a prime-boost vaccination regimen was compared to a single-shot regimen, using groups of 20 SPF broilers. Each vaccination contained 50 μg crecFib-8b/8a formulated in a 40% (wt/vol) antigen-oil-based adjuvant phase, administered i.m. Challenge was carried out i.m. with 106.2 TCID_50_ of FAdV-8a (strain 11-16629) or FAdV-8b (strain 13-18153), while negative-control birds again received PBS instead.

Blood was collected weekly from the second week of life until challenge and then at each of the following sampling time points: 3, 5, 7, and 14 dpc. Up to five birds/group were killed and necropsied at 3, 5, 7, and 14 dpc alongside individuals that died due to the infection.

Endpoints of protection included organ-BW ratios for liver, spleen, and bursa of Fabricius, the AST content in plasma, and viral loads in liver, pancreas, spleen, and bursa of Fabricius.

Statistical analysis of the data sets was carried out using the Shapiro-Wilk test together with a visual inspection of histograms and normal Q-Q plots in order to verify the normal distribution assumption. The mean values for organ-BW ratios, plasma AST levels, and viral loads in target organs of vaccinated groups were compared with the values for the negative-control group and the corresponding challenge control group via unpaired Student’s *t* test. Data sets that did not meet the normality assumptions were analyzed through pairwise comparisons with Mann-Whitney U test. In each case, *P* values of ≤0.05 were considered statistically significant. Statistical analyses were performed with SPSS version 26 (IBM SPSS Statistics; IBM Corp., Armonk, NY, USA).
