# Supplementary material for: Recombinantly Expressed Chimeric Fibers Demonstrate Discrete Type-Specific Neutralizing Epitopes in the Fowl Aviadenovirus E (FAdV-E) Fiber, Promoting the Optimization of FAdV Fiber Subunit Vaccines towards Cross-Protection in vivo
Source: Microbiol Spectr. 2022 Jan 19;10(1):e02123-21. doi: 10.1128/spectrum.02123-21 (PMC8768839; doi:10.1128/spectrum.02123-21)
Supplement: SUPPLEMENTAL FILE 1 — Supplemental material. Download SPECTRUM02123-21_Supp_1_seq12.pdf, PDF file, 0.6 MB [file spectrum02123-21_supp_1_seq12.pdf]

(a)

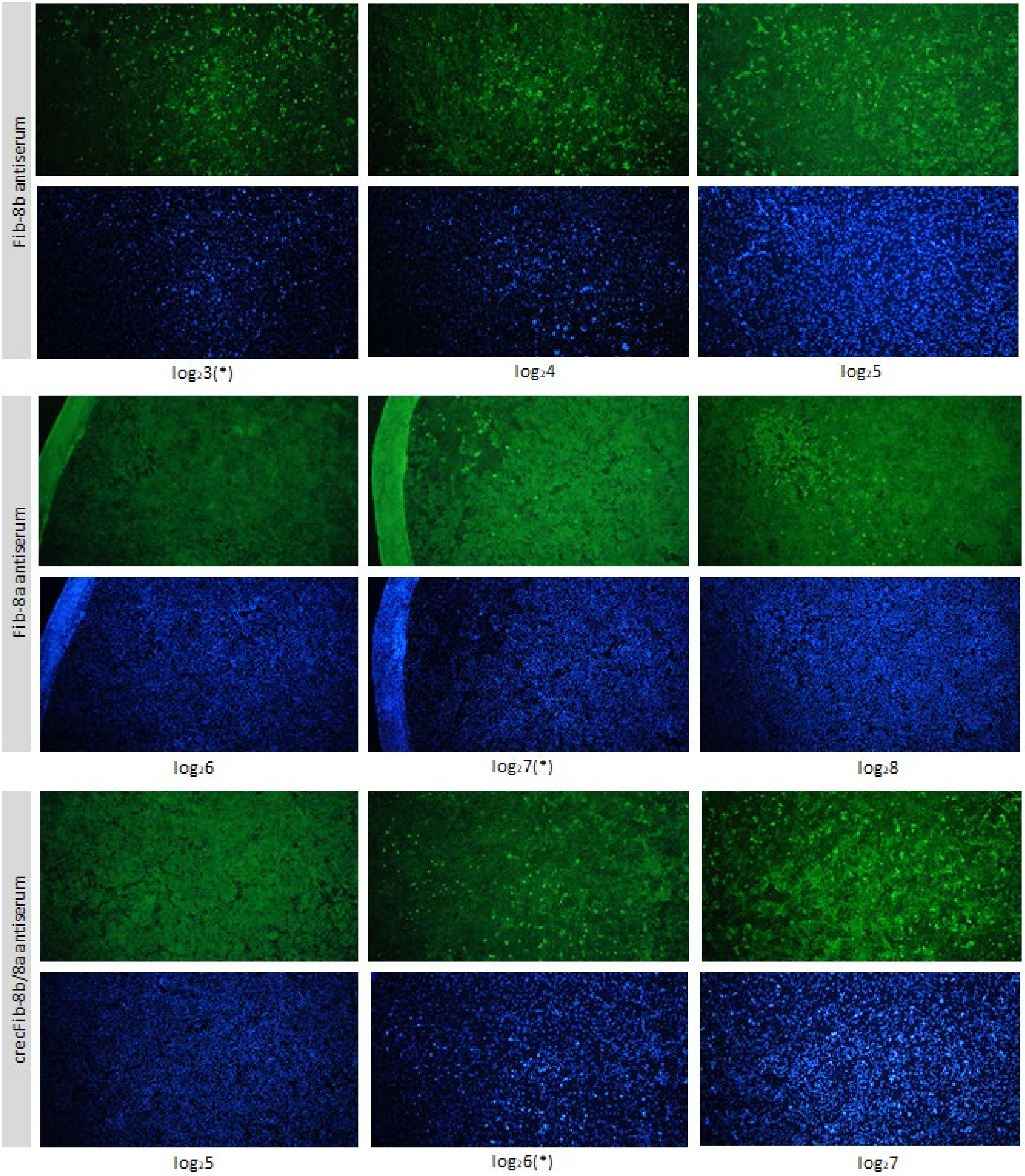
Fib-8b antiserum
log2 3(*)
log2 4
log2 5
Fib-8a antiserum
log2 6
log2 7(*)
log2 8
crecFib-8b/8a antiserum
log2 5
log2 6(*)
log2 7

(b)

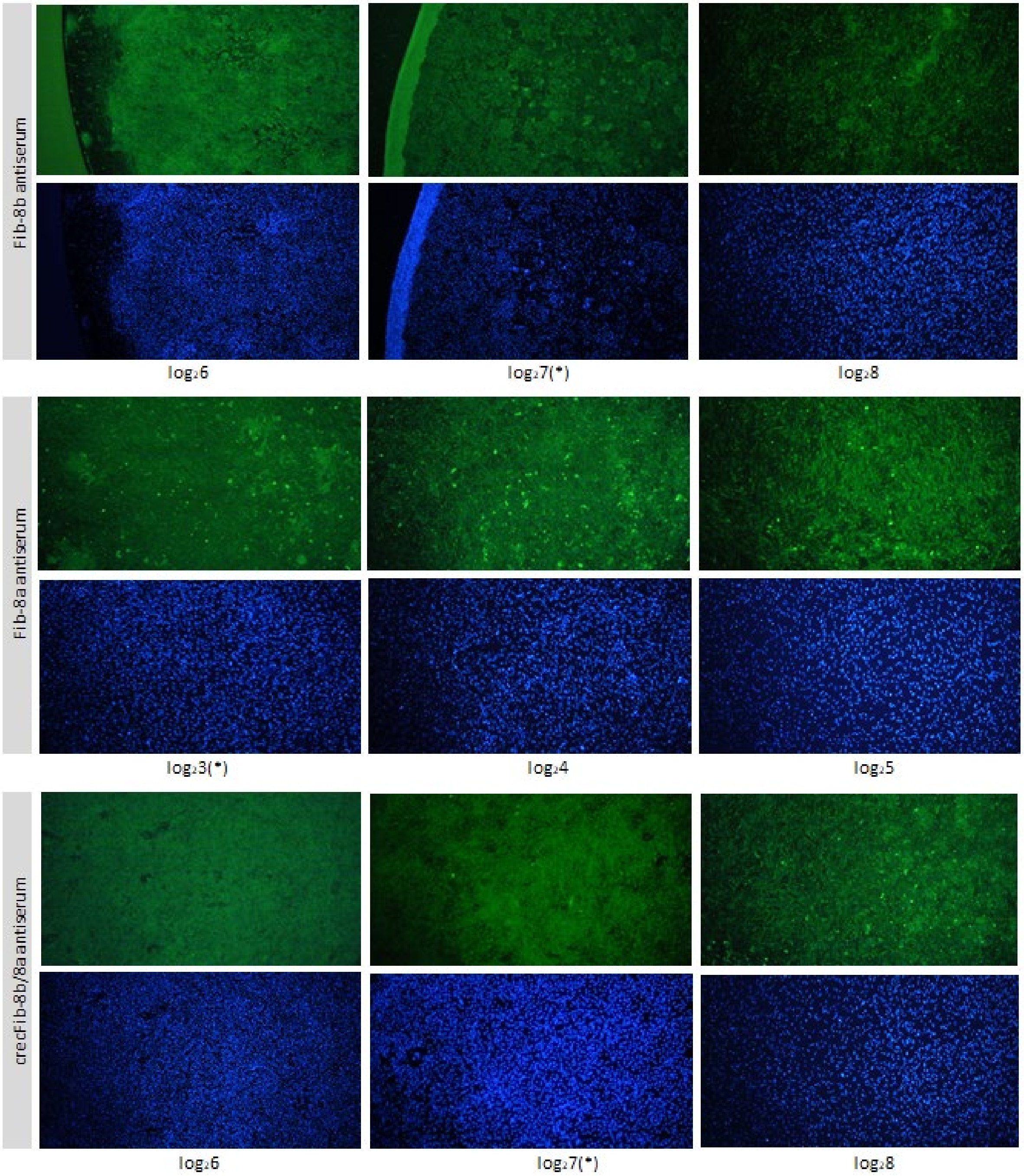
Fib-8b antiserum
log₂6
log₂7(*)
log₂8
Fib-8a antiserum
log₂3(*)
log₂4
log₂5
crecFib-8b/8a antiserum
log₂6
log₂7(*)
log₂8

(c)

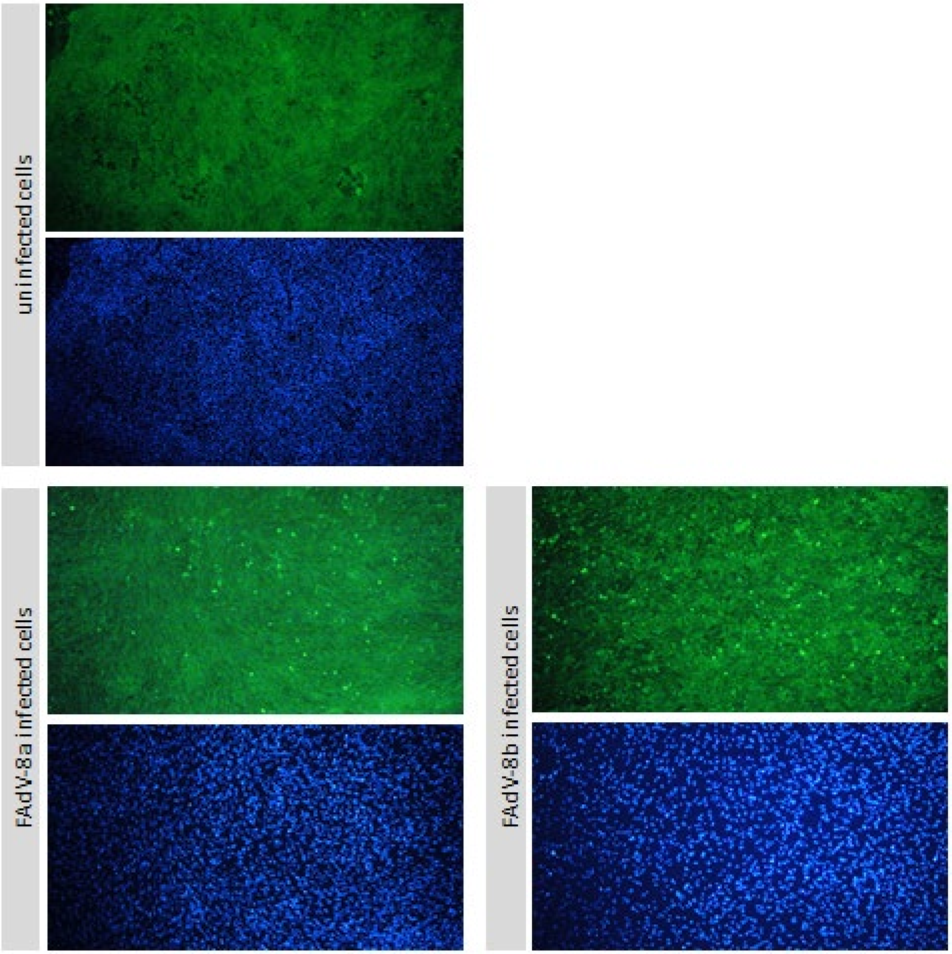

Supplemental figure S1. Immunofluorescence staining of viral particles´ internalization into CEL cells, and inhibition by different categories of fiber antisera (objective 5x). The same three antisera (indicated at the left margins), tested side-by-side against (a) FAdV-8a reference strain TR59 and (b) FAdV-8b reference strain 764. A series of three successive serum dilutions is exemplarily shown, with titers

indicated below each image. The lowest titer level at which stained viral particles (and CPE) were detected is marked by an asterisks. Identical sections of the wells are shown on top of each other for Alexa Fluor 488 and cell nuclei staining (DAPI). Panel (c) shows control wells with uninfected, FAdV-8a/TR59-infected and FAdV-8b/764-infected cells. Bar, 100 μm.

Supplemental table S1. Cloning information on the crecFib proteins of this study. Overhangs at the 5´-termini of primer sequences with the flanking vector sequence (FP1 and RP2 primers) or with the counterpart template sequence (RP1 and FP2 primers) are represented by underlined nucleotides.

Designation of chimeric construct	Fragment (position in template sequence)	Template strain (GenBank accession number)	Primer sequences	Expression vector (restriction sites used for cloning)
crecFib-8a/8b	I (nt1-1323)	TR59, FAdV-8a reference strain (KT862810)	FP1-TR59[a]: 5´-AAA CCT GTA TTT TCA GGG CAT GGC GAC CTC GAC TC-3´<br>RP1-TR59: 5´-GAT AGC TTT CCA GTC GCC CGG TGT TTG GTT GGA AA-3´	pFAST BAC HTb (*EheI/StuI*)
	II (nt1324-1569)	764, FAdV-8b reference strain (KT862811)	FP2-764: 5´- ACC GGG CGA CTG GAA AGC TAT CTC CCC GTC CTT AC-3´<br>RP2-764: 5´- GTG AGC TCG TCG ACG TAG GTT AAG GAG CGT TGG CG-3´	
crecFib-8b/8a	I (nt1-1323)	764, FAdV-8b reference strain (KT862811)	FP1-764[a]: 5´-AAA CCT GTA TTT TCA GGG CAT GGC GAC CTC GAC TC-3´<br>RP1-764: 5´-GGT ATG TGT CCA CTC GAC CAC TGG TGG GTT CAA AA-3´	pFAST BAC HTb (*EheI/StuI*)
	II (nt1324-1575)	TR59, FAdV-8a reference strain (KT862810)	FP2-TR59: 5´-AGT GGT CGA GTG GAC ACA TAC CTG CCG GTT CTC AC-3´<br>RP2-TR59: 5´- GTG AGC TCG TCG ACG TAG GTT ATG ACA CGT CCG CA-3´	

[a] Primers with identical sequence.

Supplemental table S2. Summary of immune sera used in this study, according to their specificity.

Specificity based on cross-neutralization		Category of antigen used for immunization			Source[a]
		Live FAdV	Inactivated FAdV	Recombinant protein	
FAdV-A	FAdV-1	11-7127 (MK572848)			Grafl et al. (2014)
FAdV-B	FAdV-5		340 (KC493646)<br>15-4225		Feichtner et al. (2018)<br>this study
FAdV-C	FAdV-4	KR5 (HE608152)<br><br><br>AG234 (MK572849)		 KR5 Fib-1 (HE608152)<br>KR5 Fib-2 (HE608152)	Feichtner et al. (2018)<br>Schachner et al. (2014)<br>Schachner et al. (2014)<br>Schachner et al. (2014)
	FAdV-10	C-2B (MK572851)			Feichtner et al. (2018)
FAdV-D	FAdV-2	685 (KT862805)<br>08-12809 (LN907547)<br><br>11-16628 (LN907545)			Feichtner et al. (2018)<br>GZ 68.205/0217-WF/V/3b/2016<br>GZ 68.205/0217-WF/V/3b/2016
	FAdV-3	SR49 (KT862807)			Feichtner et al. (2018)
	FAdV-9	A-2A (AF083975)			Feichtner et al. (2018)
	FAdV-11	380 (KT862812)<br>13-18966 (LN907556)	 <br><br>13-14796		Feichtner et al. (2018)<br>GZ 68.205/0217-WF/V/3b/2016<br>this study
FAdV-E	FAdV-6	CR119 (KT862808)	CR119 (KT862808)		Feichtner et al. (2018)
	FAdV-7	YR36 (KT862809)	YR36 (KT862809)		Feichtner et al. (2018)
	FAdV-8a	TR59 (KT862810)<br>11-16629 (MK572865)	TR59 (KT862810)		Feichtner et al. (2018)

	FAdV-8b	764 (KT862811)<br>13-18153 (MK572862)	764 (KT862811)		Feichtner et al. (2018)
hybrid fiber specificity	7/8a/6		13/19395 (MK572863)		Schachner et al. (2019)
	8a/8b			crecFib-8a/8b<br>crecFib-8b/8a	this study<br>this study

[a] Serum generated in the framework of published studies, or under the indicated animal trial license number.

References

Feichtner, F., Schachner, A., Berger, E., Hess, M. (2018). Development of sensitive indirect enzyme-linked immunosorbent assays for specific detection of antibodies against fowl adenovirus serotypes 1 and 4 in chickens. Avian Pathol. 47, 73-82.

Grafl, B., Prokofieva, I., Wernsdorf, P., Steinborn, R., Hess, M. (2014). Infection with an apathogenic fowl adenovirus serotype-1 strain (CELO) prevents adenoviral gizzard erosion in broilers. Vet. Microbiol. 172, 177-185.

Schachner, A., Gonzalez, G., Endler, L., Ito, K., Hess, M. (2019). Fowl adenovirus (FAdV) recombination with intertypic crossovers in genomes of FAdV-D and FAdV-E, displaying hybrid serological phenotypes. Viruses 11, 1094.

Schachner, A., Marek, A., Jaskulska, B., Bilic, I., Hess, M. (2014). Recombinant FAdV-4 fiber-2 protein protects chickens against hepatitis–hydropericardium syndrome (HHS). Vaccine 32, 1086-1092.
